# Sample size calculations for stepped wedge trials using design effects are only approximate in some circumstances

**DOI:** 10.1186/s13063-016-1359-4

**Published:** 2016-05-04

**Authors:** Karla Hemming

**Affiliations:** University of Birmingham, Edgbaston, Birmingham, B15 2TT UK

## Abstract

**Abstract:**

Estimation of sample size and power for stepped wedge cluster randomised trials can be determined by one of a number of related methods. These include exact analytical approaches, design effects or simulation. A recent paper compared the design effect to the analytical method. There were some differences between the two approaches. We show here that these differences occur because the design effect approach is only technically correct when there is an equal number of clusters crossing over at each step.

**Findings:**

The design effect for the stepped wedge cluster randomised trial is only appropriate when there is an equal number of clusters switching at each step.

## Background

Baio and colleagues [1] compare the estimated number of clusters needed in a sample size calculation for a stepped wedge cluster randomised trial (SW-CRT), between the analytical method proposed by Hussey and Hughes [2] and the design effect proposed by Woertman et al. [3]. Table 1 of the paper by Baio [1] shows that the results, whilst similar, do not exactly match between the two approaches. There may be several explanations for this. But one potentially important explanation is that the design effect proposed by Woertman is only valid when the same number of clusters crosses over at each step. When the number of clusters crossing over at each step is different, the arrangement of the cross-overs can result in different levels of power.

### Worked example

Suppose a trial is to be designed to detect a standardised mean difference of 0.25 at 80 % power and 5 % significance. Under individual randomisation a sample size in the region of 250 per arm is needed. This example is constructed to be similar to the example in Table 1 of [1] for the continuous outcome. Assume a cross-sectional SW-CRT design is to be used with 5 steps (equating to 6 measurement points) with a cluster size of 20 per measurement point and a total cluster size of 120(=6*20). For illustration we consider the case for which the intraclass correlation coefficient (ICC) is 0 (row 1 of Table 1 in [1]).

The design effect based on the formula by Woertman is:

$$ D{E}_{SW=}6\ast \frac{1+0.0\kern0.28em \left(5\ast 20+20-1\right)}{1+0.0\left(\frac{5\ast 20}{2}+20-1\right)}\ast \frac{3\left(1-0.0\right)}{2\left(5-\frac{1}{5}\right)}, $$

which is equal to 1.88 to 2 decimal places (dp). Multiplying this design effect by the number needed under individual randomisation gives 938 (approx. 1.88*250*2). Dividing this total sample size by the total cluster size 120 (=20*6) gives 7.82 (2 dp). Rounding up gives 8 clusters needed, randomised across 5 steps.

**Fig. 1:**
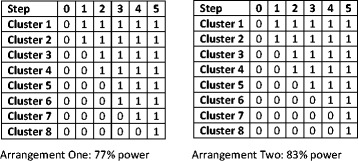
Two alternative arrangements for an SW-CRT with 5 randomisation steps and 8 clusters

However, using 8 clusters in an SW-CRT with 5 steps does not result in the same number of clusters crossing over at each step (as 8 is not a multiple of 5). So, either 1 or 2 clusters need to cross over at each step. There are, however, different ways of arranging this design. Two possible arrangements are given in Fig. 1—but there are several more. The two examples in Fig. 1 both give different values of power, even though they include 8 clusters.

Perhaps the more intuitive arrangement is to have 2 clusters randomised to each of steps 1, 2 and 3 and 1 cluster randomised to each of steps 4 and 5 (Fig. 1, arrangement one). This design, although it contains 8 clusters, results in only 77 % power, where power is computed using the analytical method described in Hussey and Hughes [2]. Of note, this is less than 80 %, which was the value used to determine the number of clusters (8).

An alternative arrangement, arrangement two in Fig. 1, has 2 clusters randomised to steps 1, 2 and 5 and 1 cluster randomised to steps 3 and 4. This arrangement provides 83 % power.

## Conclusion

In some ways the observation presented here is a technicality. But, it might have some interesting ramifications—and insights for maximising efficiency. At the very least, when using the design effect in practical applications, it is important to appreciate this difference and check that the magnitude of the differences in power is not too great.

## Abbreviations

SW-CRT: stepped wedge cluster randomised trial
